# Supplementary material for: All-Optical Control of Interfacial Polarization in MoS2/WSe2 Heterobilayers
Source: Nano Lett. 2026 Oct 8;26(41):14130–9. doi: 10.1021/acs.nanolett.6c02950 (PMC13693496; doi:10.1021/acs.nanolett.6c02950)
Supplement: Supplementary file 1 [file nl6c02950_si_001.pdf]

# *Supporting Information*

# All-Optical Control of Interfacial Polarization in $MoS_2/WSe_2$ Heterobilayers

Muhammad Sufyan Ramzan,[1] Giancarlo Soavi,[2,3] and Caterina Cocchi[1,3]

[1] *Institut für Festkörpertheorie und Optik, Friedrich-Schiller-Universität Jena, 07743 Jena, Germany*

[2]*Institut für Festkörperphysik,*[2]*Friedrich-Schiller-Universität Jena, 07743 Jena, Germany*

[3]*Abbe Center of Photonics, Friedrich-Schiller-Universität Jena, 07745 Jena, Germany*

# S1. Structural properties

The optimized in-plane lattice parameters for monolayer (ML) $MoS_2$ and $WSe_2$ are 3.166 Å and 3.294 Å, respectively. For the $MoS_2/WSe_2$ heterobilayer with equally distributed strain, the converged lattice parameter is 3.226 Å, which corresponds to a 1.90% tensile strain in the $MoS_2$ layer and a -2.06%compressive strain in $WSe_2$. In the configuration where strain is applied to $MoS_2$ (referred to as $\varepsilon_{MoS2}$ in the main text), the lattice parameter of the heterobilayer is fixed to match that of ML $WSe_2$ (3.294 Å). The atomic positions are relaxed until the forces and total energies meet the convergence criteria. An analogous procedure is adopted for the configuration where the strain is localized entirely within the $WSe_2$ layer ($\varepsilon_{WSe2}$), where the lattice parameter of the heterobilayer is fixed to that of pristine ML $MoS_2$ (3.166 Å).

# S2. Electronic properties

The $MoS_2/WSe_2$ heterobilayer exhibits a type-II band alignment, with the valence band maximum (VBM) localized on ML $WSe_2$ and the conduction band minimum (CBM) on $MoS_2$ (Figure S1). To validate our computational parameters and real-space grid choices, we reproduced the electronic structure of the $MoS_2/WSe_2$ heterobilayer under various strain configurations (Figure S1). The obtained band structures are in good agreement with corresponding VASP calculations, confirming the reliability of the real-space Octopus simulations.

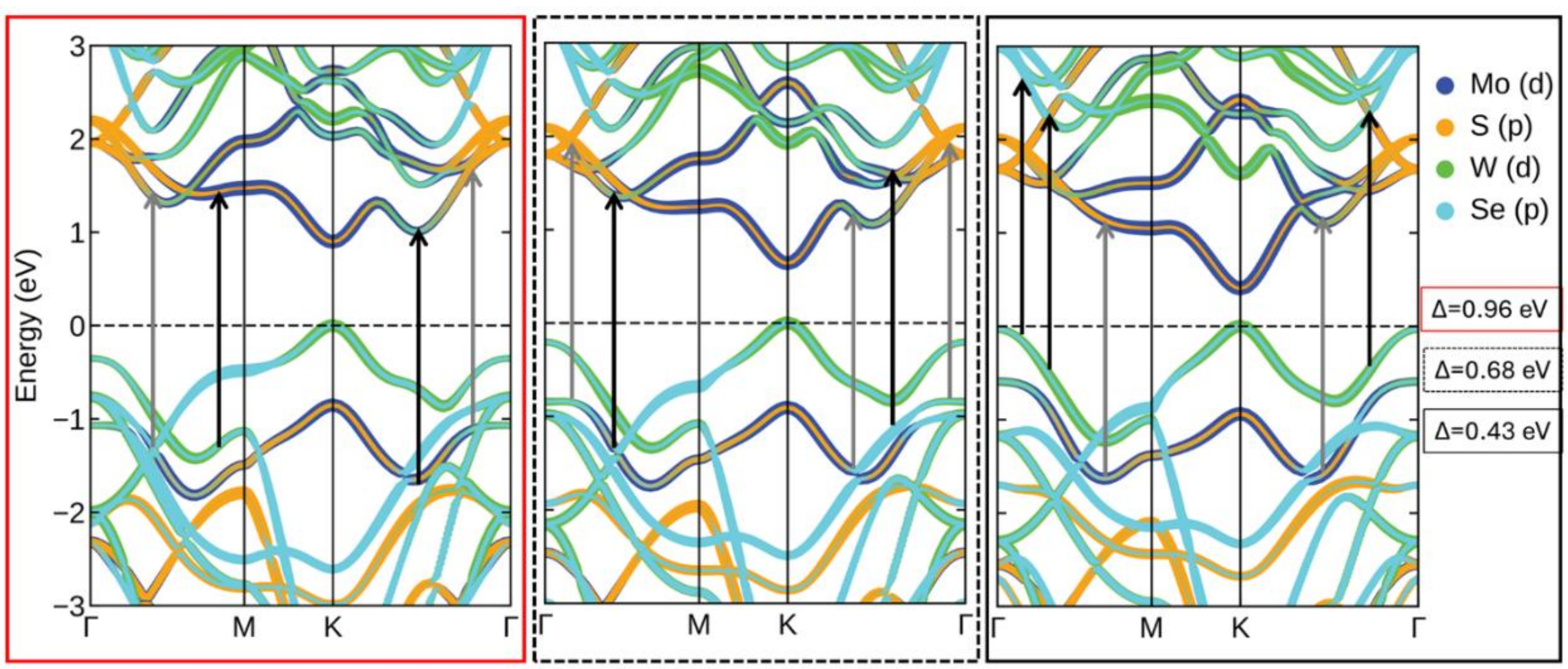


Figure S 1. Atom-projected electronic band structure of $MoS_2/WSe_2$ heterobilayer (atomic orbital contributions color-coded according to the legend) under in-plane strain applied entirely to $WSe_2$ (red solid box) and $MoS_2$ (black solid box) and equally distributed between both layers (black dashed box). These calculations were performed with VASP, using the PBE functional and neglecting spin-orbit coupling. The Fermi-level is set to 0 eV and the fundamental band gaps Δ of each configuration are reported in the legend. Black and grey arrows indicate the electronic transitions contributing with large (>10%) and moderate (<10%) weight to the primary optical resonance at 2.75 eV. It is worth noting that in all three configurations there is no contribution coming from direct transitions at K.

# S3. Laser pulse parameters and Fluence

The laser field is introduced through a time-dependent vector potential of the form

$$A(t) = A_0\, g(t) \cos\, [\omega(t - t_0)] \quad (S1)$$

here $A_0$is the vector-potential amplitude, $\omega$ is the carrier frequency, $t_0$is the pulse center (in our simulations $t_0 = \ 8fs$), and the Gaussian envelope, $g(t)$, is described as:

$$g(t) = \exp\left[-\frac{(t - t_0)^2}{2\tau_0^2}\right] \quad (S2)$$

The corresponding electric field $E(t)$, the instantaneous laser intensity, and the peak intensity is calculated as:

$$E(t) = -\frac{dA(t)}{dt} \qquad (S3)$$

$$I(t) = \frac{c}{8\pi}|E(t)|^2 \quad (S4)$$

The energy fluence (F), defined as the incident laser energy per unit area, is obtained by integrating instantaneous intensity over pulse time:

$$F = \int I\,(t)\,dt \approx \sum_i I\,(t_i)\Delta t \qquad (S5)$$

where $t_i$ denotes the $i$-th time step and $\Delta t$ is the temporal resolution of the simulation (2.41 as). Thus, the fluence accounts for the complete temporal profile of the pulse rather than being estimated simply as $I_{\text{peak}}$multiplied by a nominal pulse duration.

It is worth highlighting that the full width at half maximum (FWHM) of laser incorporated in the Octopus simulations is narrower due to squaring of envelope (Eq. S4). Therefore, the Gaussian envelope used here, $\tau_0 = 2$ fs and $t_0 = 8$ fs would have a FWHM as:

$$\text{FWHM}_A = 2\sqrt{2\ln 2}\ \tau_0 = \ 4.71\ fs,$$

while the FWHM of the corresponding squared Gaussian envelops is

$$\text{FWHM}_I = 2\sqrt{\ln 2}\ \tau_0 = \ 3.33\ fs.$$

Table S1. Time-integrated intensity (Int. Intensity) and corresponding fluence reported by the Octopus code in atomic units for each laser intensity, together with their converted values in physical units.

| **Peak intensity ($W/cm^2$)** | **Int. Intensity (a.u.)** | **Fluence (a.u.)** | **Fluence ($mJ/cm^2$)** |
|---|---|---|---|
| $1.0 \times 10^{10}$ | $1.149 \times 10^{-4}$ | $2.110 \times 10^{-5}$ | 0.018 |
| $1.0 \times 10^{11}$ | $1.149 \times 10^{-3}$ | $2.110 \times 10^{-4}$ | 0.179 |
| $5.0 \times 10^{11}$ | $5.750 \times 10^{-3}$ | $1.055 \times 10^{-3}$ | 0.895 |
| $1.0 \times 10^{12}$ | $1.150 \times 10^{-2}$ | $2.109 \times 10^{-3}$ | 1.790 |
| $5.0 \times 10^{12}$ | $5.750 \times 10^{-2}$ | $1.055 \times 10^{-2}$ | 8.950 |

# S4. Nonlinear optical response

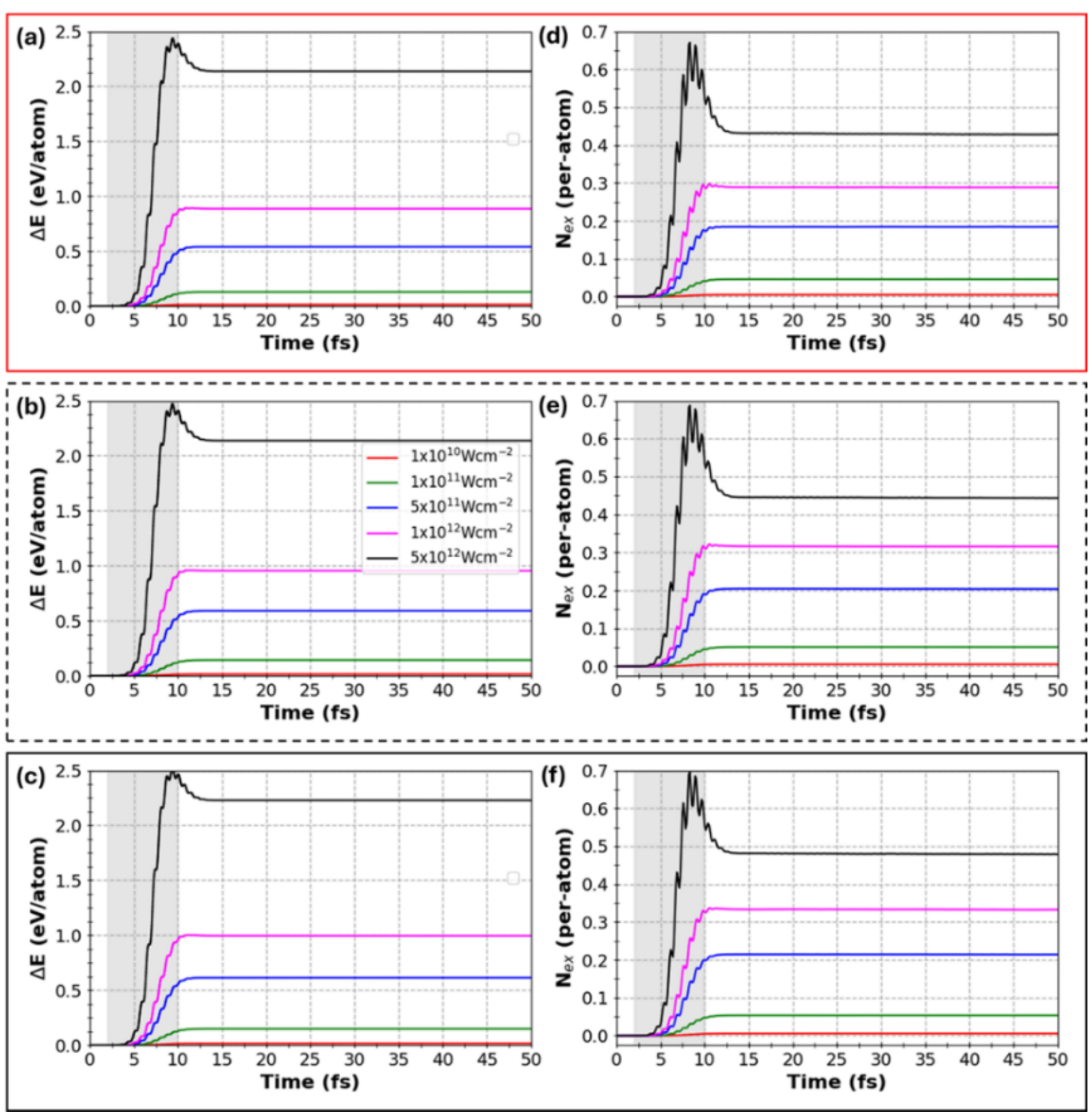


Figure S 2. (a) - (c) Time evolution of the electronic energy uptake (ΔE) and (d) - (f) number of excited electron per atom ($N_{ex}$) under varying laser intensities corresponding to $1{\times}10^{10}$ $Wcm^{-2}$ (red), $1{\times}10^{11}$ $Wcm^{-2}$ (green), $5{\times}10^{11}$ $Wcm^{-2}$ (blue), $1{\times}10^{12}$ $Wcm^{-2}$ (magenta), and $5{\times}10^{12}$ $Wcm^{-2}$ (black). Results obtained for the $MoS_2/WSe_2$ heterobilayer with in-plane strain applied to $WSe_2$ and $MoS_2$ monolayers are reported in the red and black solid boxes, respectively, while those computed with equally distributed strain are in the black dashed box. The grey shaded regions mark the window of laser irradiation.

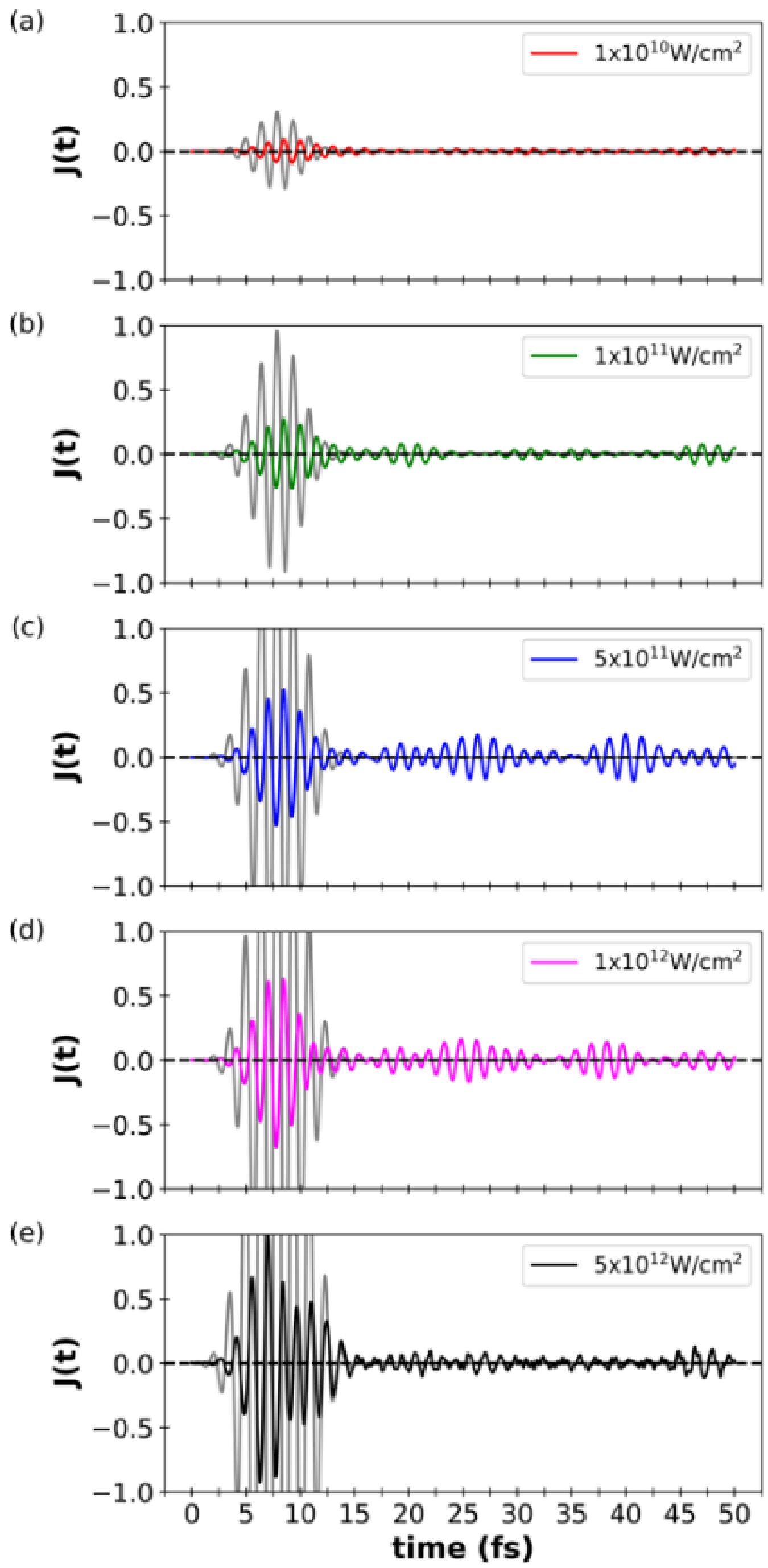


Figure S 3. Time evolution of current densities J(t) in $MoS_2/WSe_2$ heterobilayer with average strain, under laser pulses of increasing peak intensities: (a) $1\times10^{10}$ $Wcm^{-2}$, (b) $1\times10^{11}$ $Wcm^{-2}$, (c) $5\times10^{11}$ $Wcm^{-2}$, (d) $1\times10^{12}$ $Wcm^{-2}$, and (e) $5\times10^{12}$ $Wcm^{-2}$, shown in grey in the background.

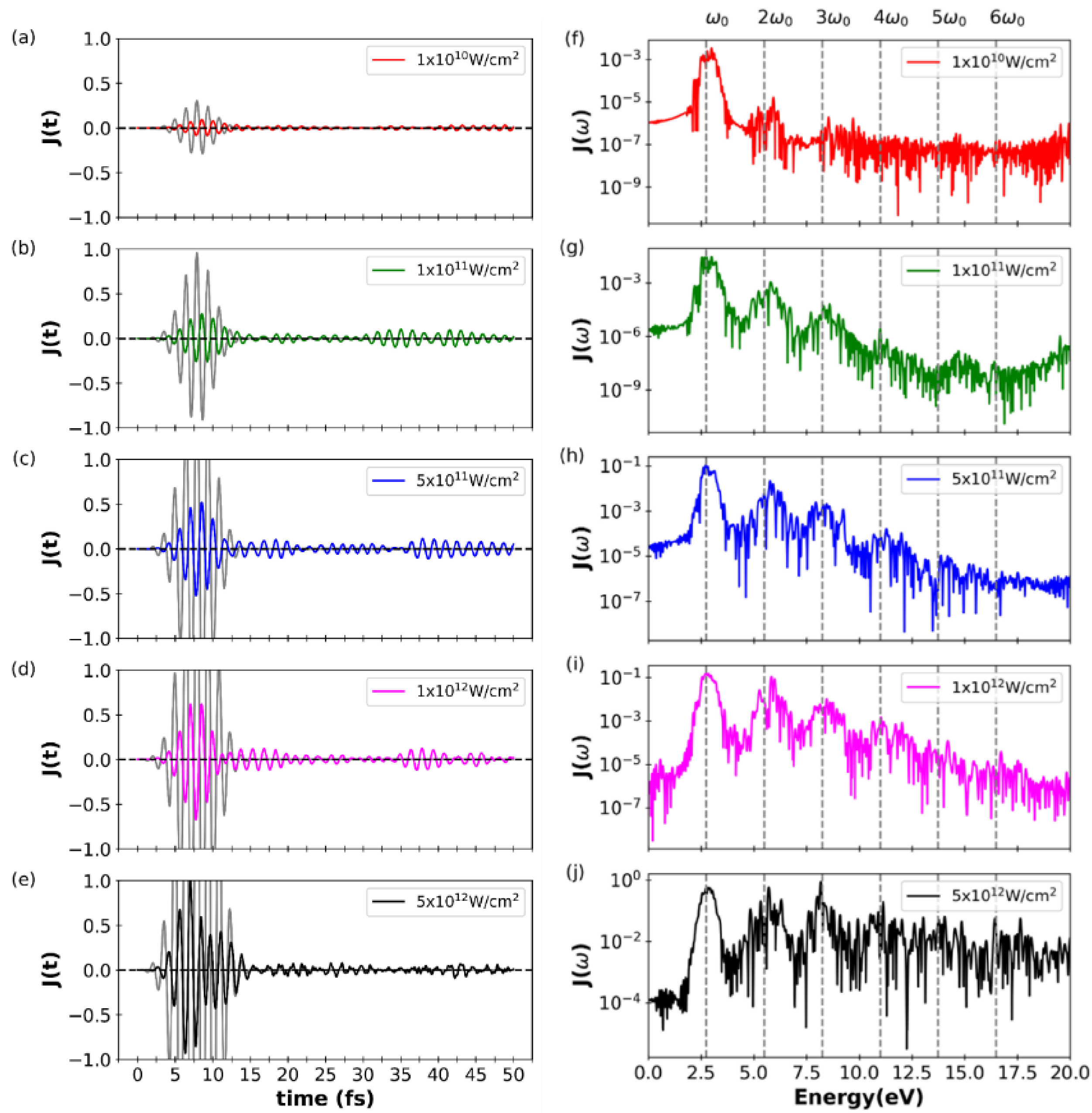


Figure S 4. Time evolution of current densities J(t) in $MoS_2/WSe_2$ heterobilayer with strain applied exclusively to the $MoS_2$ ML, under laser pulses of increasing peak intensities: (a) $1\times10^{10}$ $Wcm^{-2}$, (b) $1\times10^{11}$ $Wcm^{-2}$, (c) $5\times10^{11}$ $Wcm^{-2}$, (d) $1\times10^{12}$ $Wcm^{-2}$, and (e) $5\times10^{12}$ $Wcm^{-2}$, shown in grey in the background. (f) – (j) Corresponding high-harmonic spectra J(ω), obtained via Fourier transformation of the induced current densities in (a-e). The fundamental carrier frequency ($\omega_0$) and its harmonics are indicated by vertical dashed lines.

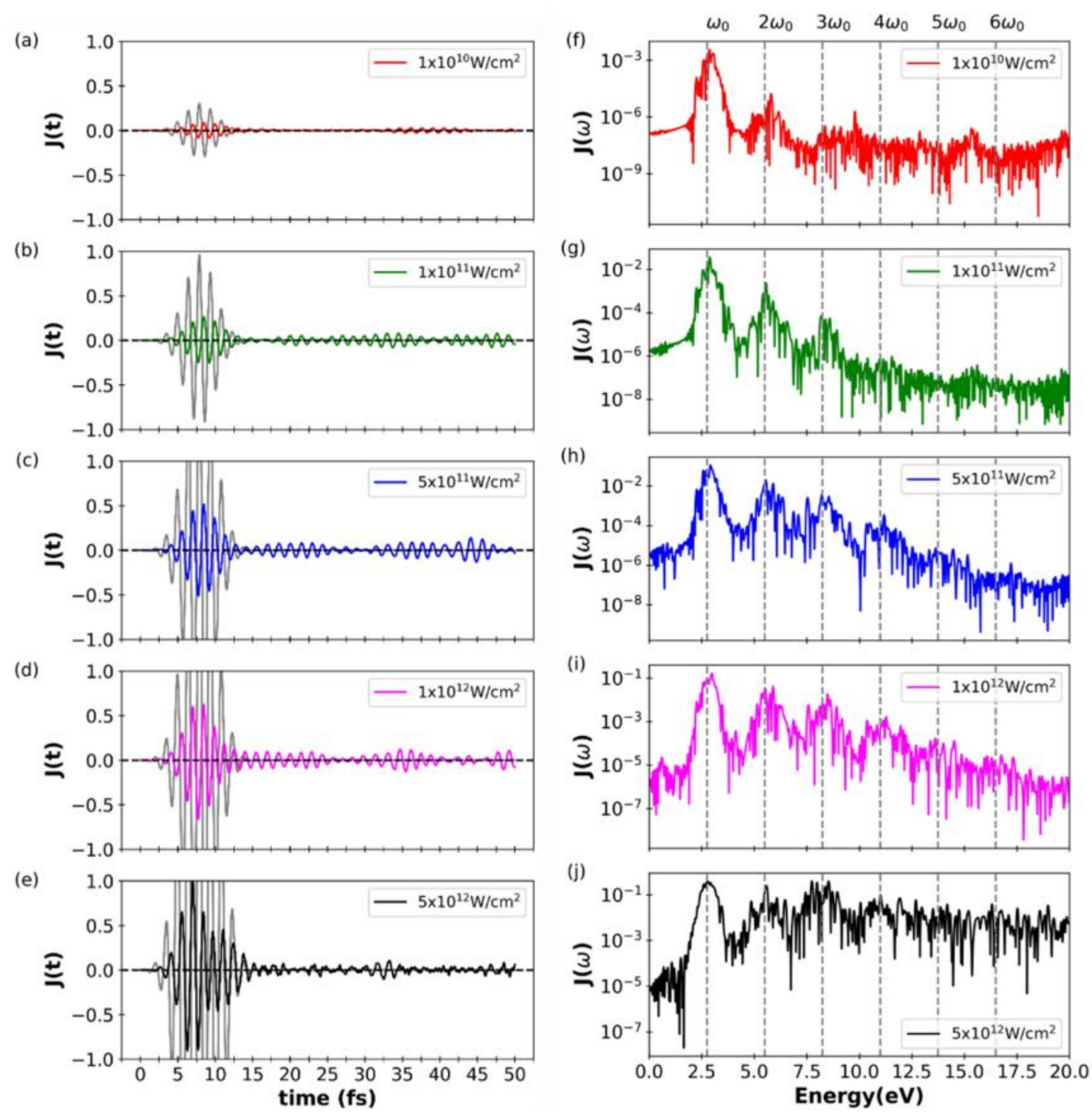


Figure S 5. Time evolution of current densities J(t) in $MoS_2/WSe_2$ heterobilayer with strain applied exclusively to the $WSe_2$ ML, under laser pulses of increasing peak intensities: (a) $1\times10^{10}$ $Wcm^{-2}$, (b) $1\times10^{11}$ $Wcm^{-2}$, (c) $5\times10^{11}$ $Wcm^{-2}$, (d) $1\times10^{12}$ $Wcm^{-2}$, and (e) $5\times10^{12}$ $Wcm^{-2}$, shown in grey in the background. (f) – (j) Corresponding high-harmonic spectra J(ω), obtained via Fourier transformation of the induced current densities in (a-e). The fundamental carrier frequency ($\omega_0$) and its harmonics are indicated by vertical dashed lines.

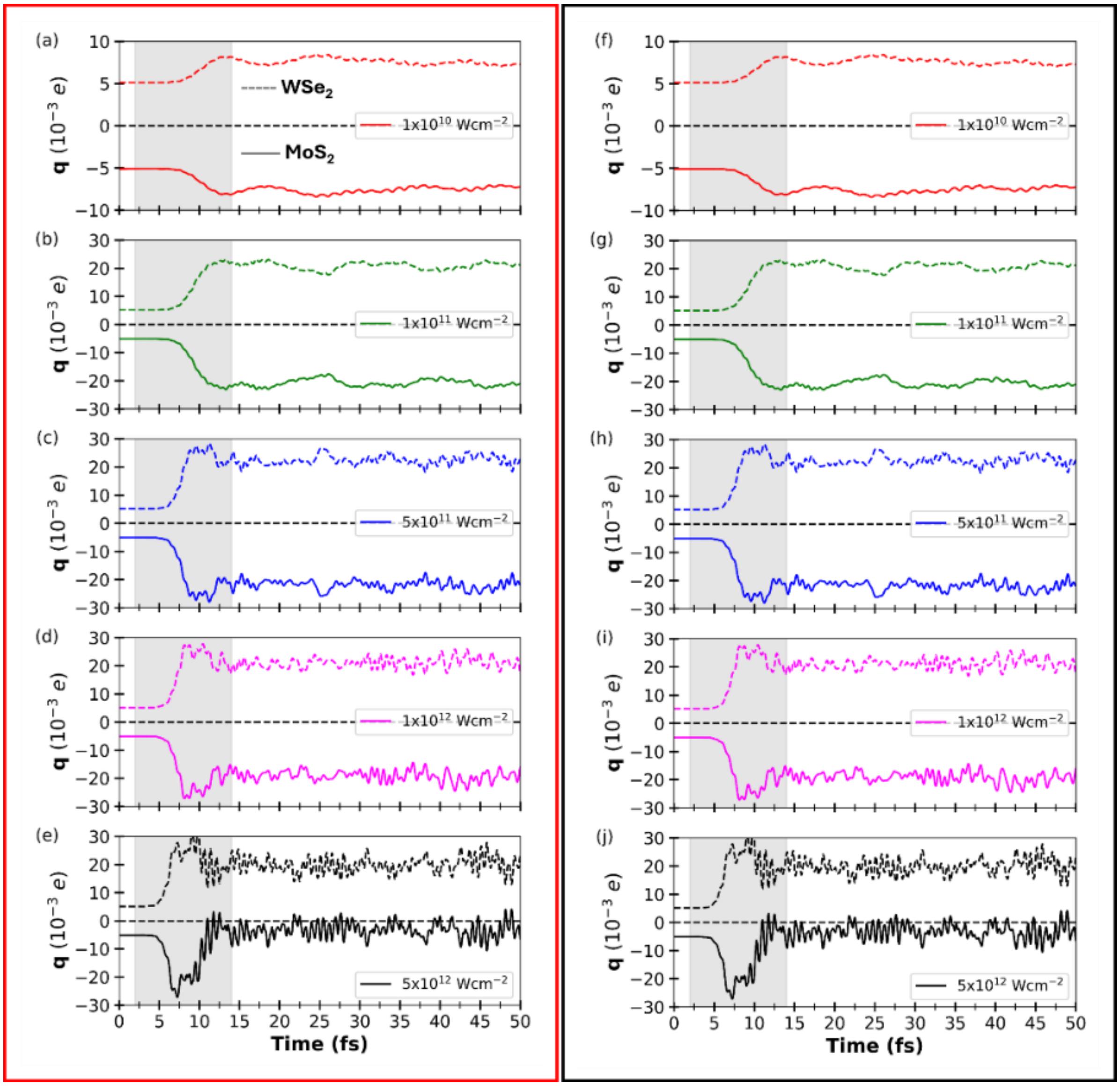


Figure S 6. Time evolution of the layered-resolved atomic charges under the laser intensities of $1\times10^{10}$ $Wcm^{-2}$ (red), $1\times10^{11}$ $Wcm^{-2}$ (green), $5\times10^{11}$ $Wcm^{-2}$ (blue), $1\times10^{12}$ $Wcm^{-2}$ (magenta), and $5\times10^{12}$ $Wcm^{-2}$ (black) for heterobilayer with strain on (a - e) ML $WSe_2$ and (f - j) ML $MoS_2$. The grey area denotes the time window of laser irradiation.

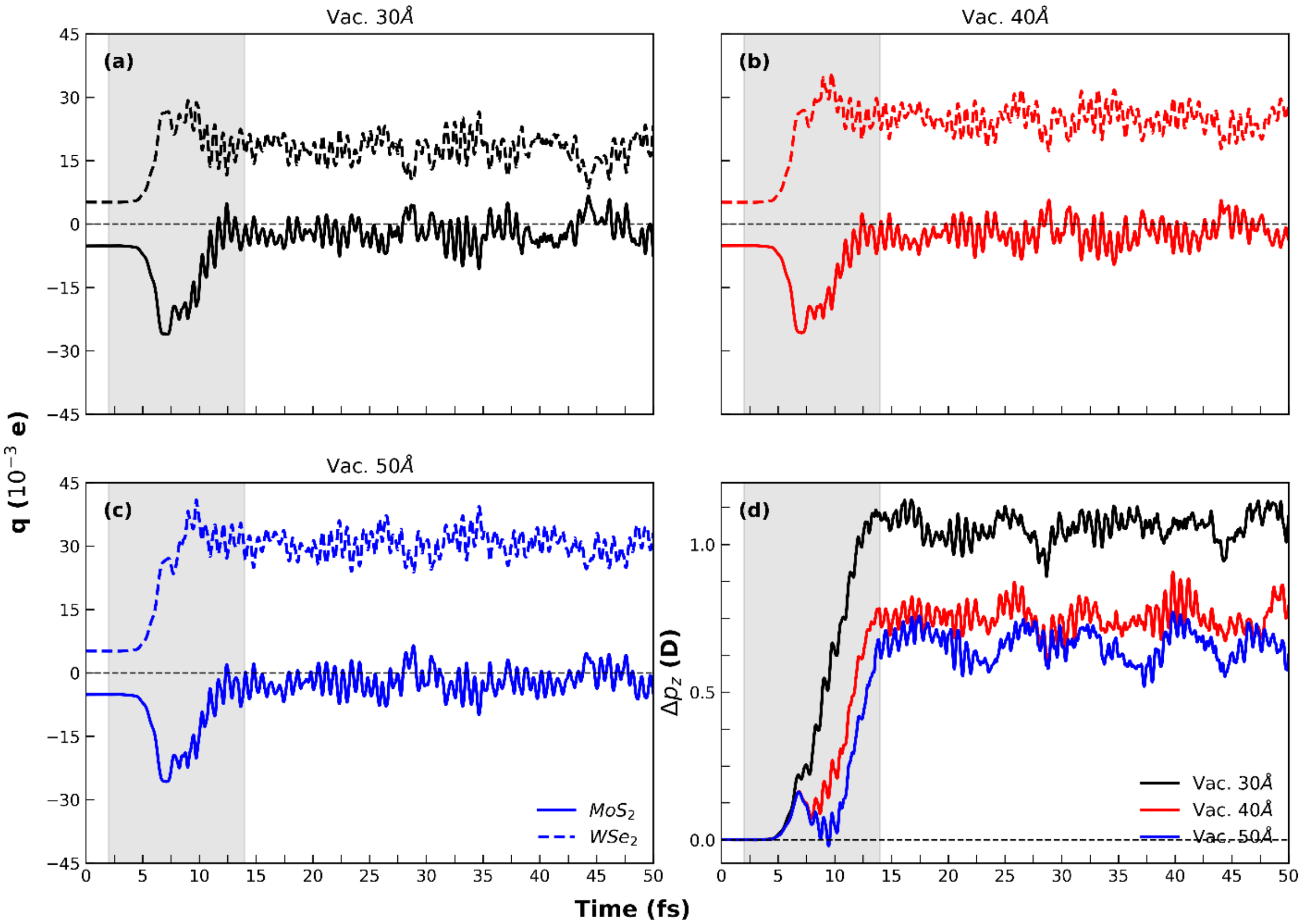


Figure S 7. Time evolution of the layered-resolved atomic charges (a) – (c) and out-of-plane dipole (d) under laser intensity of 5×10$^{12}$ Wcm$^{-2}$ for the heterobilayer with strain distributed to both layers with a minimum vacuum layer of 30 Å (black), 40 Å (red), and 50 Å (blue) thickness. The grey shaded area indicates the time window of laser irradiation.

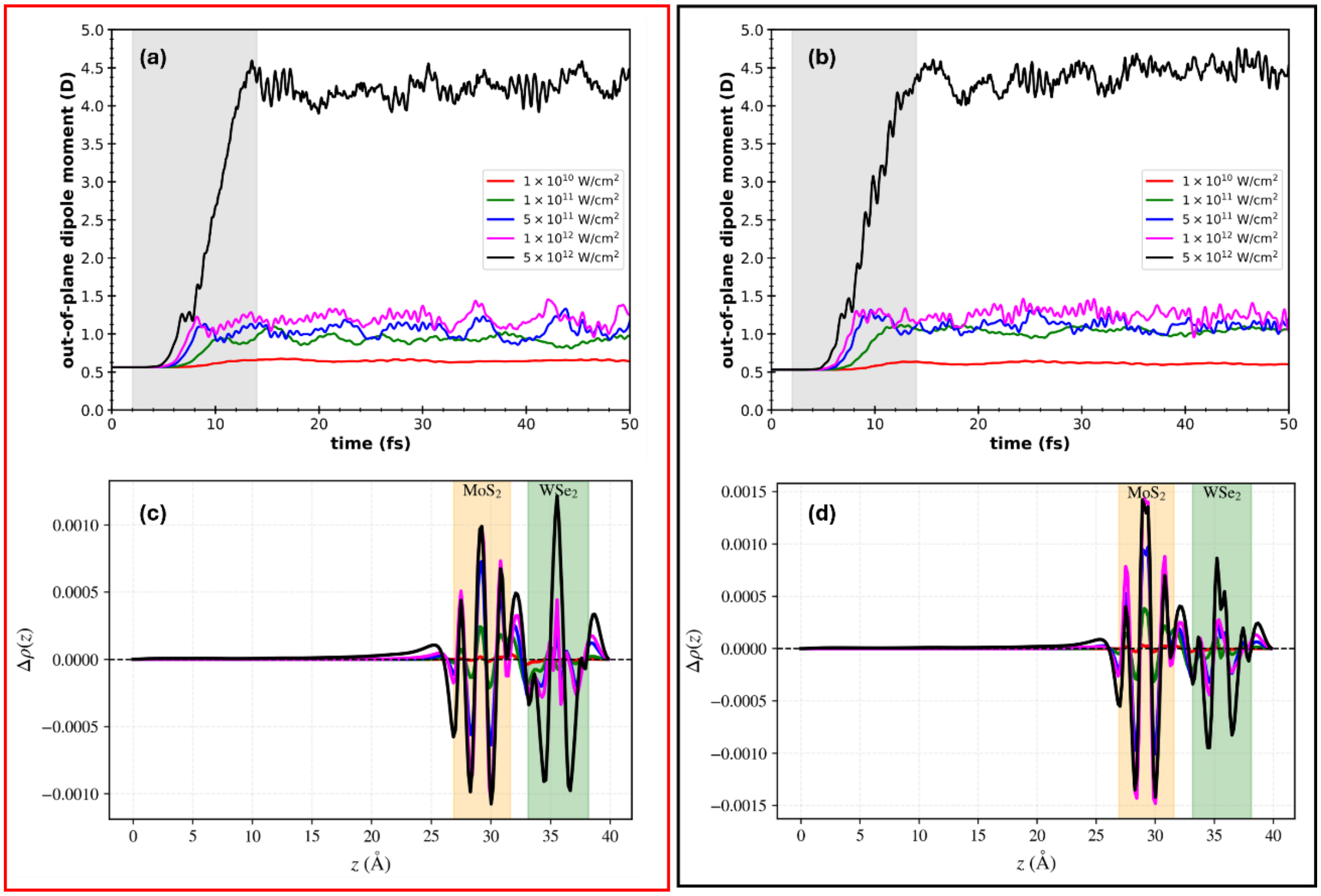


Figure S 8. (a – b) Time evolution of the out-of-plane dipole and (c – d) planar-averaged induced charge density different, $\Delta\rho(z)$, under increasing laser intensities: $1\times10^{10}$ $Wcm^{-2}$ (red), $1\times10^{11}$ $Wcm^{-2}$ (green), $5\times10^{11}$ $Wcm^{-2}$ (blue), $1\times10^{12}$ $Wcm^{-2}$ (magenta), and $5\times10^{12}$ $Wcm^{-2}$ (black) for the heterobilayer with strain applied to monolayer $WSe_2$ (left) and monolayer $MoS_2$ (right). The grey shaded area indicates the time window of laser irradiation, while orange and green shaded areas correspond to the regions occupied by $MoS_2$ and $WSe_2$ layers, respectively.
